# Association between ultra-processed food intake, diet quality, and cardiovascular risk factors among adolescents in Antioquia, Colombia

**DOI:** 10.3389/fpubh.2025.1710967

**Published:** 2025-12-17

**Authors:** Laura Castrillón-Ruiz, Alejandro Estrada-Restrepo, Gustavo Cediel, Diana Cárdenas-Sánchez, Jacqueline Barona-Acevedo, Juan C. Aristizábal

**Affiliations:** 1 School of Nutrition and Dietetics, University of Antioquia, Medellín, Colombia; 2 Research group of Toxinology, Food and Therapeutic Alternatives, School of Microbiology, University of Antioquia, Medellin, Colombia; 3 Physiology and Biochemistry Research Group-PHYSIS, Faculty of Medicine, University of Antioquia, Medellín, Colombia

**Keywords:** adolescents, ultra-processed foods, NOVA classification, heart disease risk factors, public health

## Abstract

**Objective:**

This study aimed to analyze the relationship between ultra-processed food (UPF) intake, diet quality, and cardiovascular risk factors (CRFs) among adolescents in Antioquia, Colombia.

**Methodology:**

This study is a cross-sectional secondary analysis of adolescents who participated in food and nutritional surveys in Antioquia. The NOVA classification was used to identify the intake of UPF based on 24-h dietary recalls. The analysis focused on the contribution of UPF intake to overall dietary kilocalories, macronutrients, and fiber content. The CRFs included excess body weight, abdominal obesity, and alterations in blood lipid and glucose levels. Statistical analyses included multiple linear regression models, multivariate binary logistic regression, and odds ratios (ORs) with 95% confidence intervals.

**Results:**

The analysis focused on 402 adolescents (mean age 14.1 ± 1.9 years). UPF intake accounted for 17.6 ± 1.9% of total dietary kilocalories, with a higher percentage in urban areas (19.0% vs. 9.7%; *p* = 0.002) and among those in the middle socioeconomic stratum (23.0% vs. 16.6%; *p* = 0.006). UPF consumption was associated with total kilocalories, total fat and saturated fat intake, and decreased fiber intake (*p* < 0.001). Adolescents in the highest quartile of UPF intake showed higher blood glucose levels (3.13 mg/dL; *p* < 0.05), although no significant associations were found with other CRFs.

**Conclusion:**

UPF intake was associated with diet quality deterioration and increased blood glucose levels. High UPF intake among adolescents in urban areas of Antioquia, particularly within the middle socioeconomic stratum, poses a potential health risk that warrants further monitoring.

## Introduction

The consumption of ultra-processed foods (UPFs) has been associated with overweight, obesity, and other cardiovascular risk factors (CRFs) in adolescents (1, 2). The global prevalence of obesity among adolescents has quadrupled since 1990 and has become a health concern (3, 4). In Colombia, the National Nutrition Status Surveys indicate that between 2010 and 2015, the prevalence of overweight among adolescents increased from 12.3 to 13.9%, while obesity rose from 2.8 to 4.0% (5). A similar trend was observed between 2015 and 2019 in Antioquia, which is the second most populated region in Colombia (6).

The rise in overweight, obesity, and CRFs among adolescents is likely related to the ongoing food transition in Colombia, with an increase in UPF intake and a decrease in minimally processed foods (6). In fact, a recent study reported that UPF intake increased from 2005 to 2015 among children and adolescents in Colombia (7). Additionally, national data collected in 2005 showed that the prevalence of soft drink intake was 26.4%, while this prevalence increased to 89.4% in 2010 (8, 9). On the other hand, in Colombian adolescents between 2010 and 2015, fruit and vegetable intake decreased by 6.7% and 3.2%, respectively, deteriorating the quality of their diet (5, 8).

A high uptake of UPF has been linked to deterioration in diet quality and the presence of CRFs (10–13). Some potential mechanisms contributing to diet deterioration include higher consumption of free sugars, saturated and trans fats, and sodium, along with reduced intake of fiber, protein, vitamins, and minerals (13, 14). Generally, UPFs are highly palatable and energy-dense that produce less satiety than unrefined whole foods, promoting overconsumption (13–15). Therefore, chronic consumption of UPF may contribute to overweight and obesity. Increased body fat, particularly visceral fat, promotes a pro-inflammatory state and insulin–resistance, leading altered glucose (hyperglycemia) and lipid metabolism (dyslipidemia) (16–18). However, some controversy persists, as some literature reviews report mixed findings regarding the deleterious effects of UPF intake in children and adolescents (11, 19). This suggests that other factors, such as environment, genetics, and lifestyle, may play a role in the effects of UPF on human health. Given the food transition taking place in Colombia, where adolescents have the highest UPF intake (5–8), this study aimed to analyze the association between UPF intake and diet quality, excess body weight, abdominal obesity, and alterations in blood lipid and glucose levels among adolescents in Antioquia, Colombia.

## Methods

This study is a cross-sectional secondary analysis of adolescents who participated in the Food and Nutritional Security Profile of Medellín 2015 (Medellín Profile) and Food and Nutritional Profile of Households of Antioquia 2019 (Antioquia Profile) surveys. These surveys collected information from representative samples of adolescents from the second most populous state in Colombia (Antioquia, with more than six million people) (6, 20). The sample designs for both surveys were similar, with stratified random sampling, representation by urban and rural areas, and inclusion of different age groups. The surveys (Medellín Profile and Antioquia Profile) were conducted by the same researchers from the University of Antioquia, following the same protocols and procedures for anthropometric, dietary, and blood sample collection. In addition, biochemical analyses for both surveys were performed in the Clinical Laboratory of the School of Microbiology at the University of Antioquia, following the same automated and standardized protocols and methods (6, 20). The Medellín Profile was approved by the Bioethics Committee of the Faculty of Dentistry of the University of Antioquia (Act no. 1 dated 27 February 2015), and the Antioquia Profile was approved by the Ethics Committee of the Faculty of Medicine of the University of Antioquia (Act No. 12 dated 23 August 2018). Both surveys were conducted in accordance with the Declaration of Helsinki of 2008, and informed consent was obtained from all participants and their caregivers.

### General characteristics of the participants

Variables such as age, sex, geographic location (urban or rural), physical activity level (sedentary, moderate, or vigorous), and socioeconomic stratum (low or middle) were analyzed. In Colombia, socioeconomic stratum classification is based on the physical characteristics of the property and the surrounding environment in which a person resides. Factors such as housing materials (for facades and roofs), the size of front yards, roads, and sidewalks were also considered. Strata levels were classified as follows: strata 1 and 2 are considered low, strata 3 and 4 are considered medium, and strata 5 and 6 are considered high. Adolescents with complete information on sociodemographic, anthropometric, physical activity, biochemical, and food intake variables were included in the analysis. Athletes and pregnant or breastfeeding adolescents were excluded from the study.

### Food intake analysis

All 24-h dietary recalls were analyzed to quantify UPF intake. The 24-h dietary recall was applied using the multiple-pass method and randomly distributed across all weekdays and weekends to capture daily variability. A second recall was conducted in 25% of the sample, ensuring that it was used on a different day of the week from the first recall. The interviews were conducted by trained and standardized dietitians supervised by an expert nutritionist and dietitian who checked the quality of the data at the collection point. To minimize the likelihood of underreporting, the interviewers used validated food models, and caregivers or food preparers participated in the interviews to improve the accuracy of the reported quantities. In both surveys (Medellín and Antioquia), only fully completed 24-h recalls were accepted during the data collection process.

The reported foods were grouped into four NOVA classification groups: 1. Natural or minimally processed foods; 2. Culinary ingredients; 3. Processed foods; and 4. Ultra-processed food products (21). Reported mixed dishes were broken down into dietary ingredients at the time of collection by trained and standardized dieticians and then classified using the NOVA proposal. For each NOVA group, the dietary contributions of energy, protein, carbohydrates, total fats, saturated fats, and grams of fiber per 1,000 kilocalories were calculated using the Evaluation of Dietary Intake software (version 5) (EVINDI5 for its acronym in Spanish). UPF information not available in EVINDI5 was complemented by data from the United States Department of Agriculture Food Composition Table and UPF nutrition labels.

### Nutritional status and biochemical variables

Information regarding body weight, height, and waist circumference was used to assess the nutritional status of adolescents. Body mass index (BMI) and BMI-for-age Z score (BMI/A) were calculated using Anthro Plus software from the World Health Organization (WHO). BMI/A was classified using the WHO guidelines (22). Given the low proportion of adolescents with thinness and obesity in this study, two grouping categories were created. The category “excess body weight” gathers overweight and obese adolescents, and the category of “not excess body weight” includes those with thinness and normal body weight. Waist circumference was used to determine the presence of abdominal obesity in adolescents using the International Diabetes Federation (IDF) cutoff points (23).

Blood lipid and glucose levels were used to evaluate the presence of CRFs. Alterations in triglyceride, high-density lipoprotein cholesterol (HDL-C), and glucose levels were determined using the IDF cutoff points (23). Cut-off points for altered values of total cholesterol and low-density cholesterol (LDL-C) were established according to the Integrated Guidelines of the Expert Panel for Cardiovascular Health and Risk Reduction in Children and Adolescents of the United States (24).

### Statistical analysis

The normality of the data distribution was assessed using the Kolmogorov–Smirnov test. The results are presented as mean ± standard deviation or median and interquartile range (IQR) according to the distribution of the data. The adolescents’ data were grouped into UPF consumption quartiles to better control for the variability in UPF intake in each quartile. Multiple linear regression models were used to evaluate the UPF caloric intake associations with BMI, waist circumference, blood lipids, and glucose concentrations; anthropometry and blood lipid variables were transformed using Box-Cox. Binary logistic regression models were used to evaluate the association between UPF caloric intake and excess body weight, abdominal obesity, and alterations in total cholesterol, triglycerides, HDL-C, and LDL-C; logistic regression models were evaluated using Hosmer and Lemeshow tests. Both models were adjusted for age (years), sex, socioeconomic stratum, geographic area, physical activity level (sedentary, active, or vigorous), and total calorie intake. Moreover, the variance inflation factor was less than five for all models when multicollinearity was evaluated. The last analysis did not include alterations in blood glucose levels, given the low frequency of this condition in the analyzed population. A *p*-value of <0.05 was considered statistically significant. IBM Statistical Package for Social Sciences (SPSS), version 29.0, was used for statistical analysis.

## Results

Four hundred and two adolescents (52.5% men) were included in the study, with a mean age of 14.1 ± 1.9 years. Most adolescents were inhabitants of the urban areas of Antioquia (89.3%) and belonged to low socioeconomic strata (83.3%). Many adolescents had a normal body weight (80.5%) and did not have abdominal obesity (92.2%). Notably, low levels of HDL-C and high concentrations of triglycerides were found in 35.6% and 14.6% of participants, respectively. Table 1 describes the participants’ general characteristics and biochemical variables.

**Table 1:** General characteristics of the adolescents from Antioquia.

Variables	Value
Sex (male %)	52.5
Residential area (urban %)	89.3
Socioeconomic stratum (low %)	83.3
Age (years)	14.1 ± 1.9
Body weight (kilograms)	50.6 ± 13.1
Height (centimeters)	156.9 ± 10.4
BMI (kilograms/meters^2^)	19.7 (17.6–21.8)
BMI classification (excess body weight %)	19.6
Waist circumference (centimeters)	68.9 (64.5–74.6)
Abdominal obesity (%)	7.8
Total cholesterol (mg/dl)	150.4 (131.7–170.3)
High total cholesterol proportion (%)	7.8
HDL cholesterol (mg/dl)	44.0 (38.0–51.3)
Low HDL cholesterol proportion (%)	35.6
LDL cholesterol (mg/dl)	83.5 (68.7–102.5)
High LDL cholesterol proportion (%)	6.6
Triglycerides (mg/dl)	93.0 (70.5–128.0)
High triglyceride proportion (%)	14.6
Blood glucose (mg/dl)	83.5 ± 8.2
High blood glucose proportion (%)	2.3

Data are presented as percentages, mean ± standard deviation, or median and interquartile range in parentheses, according to the nature of the variable and distribution of the data. BMI: Body mass index, LDL: Low-density lipoprotein, HDL: High-density lipoprotein. BMI classification was performed using World Health Organization guidelines (22), and high triglycerides and blood glucose and low HDL cholesterol were classified using the International Diabetes Federation criteria (23). High total cholesterol, LDL cholesterol, and waist circumference (abdominal obesity) were classified according to the guidelines of the Expert Panel for Cardiovascular Health and Risk Reduction in Children and Adolescents (24).

### Ultra-processed food product intake and diet quality

Adolescents’ mean energy intake was 2025 kilocalories, with 17.6% (IR = 8.3–29.3) coming from the UPF (Table 2). The UPF intake was higher in adolescents from urban than rural areas (19.0% vs. 9.7%; *p* = 0.002) and from medium than low socioeconomic strata (23.0% vs. 16.6%; *p* = 0.006). There were no significant differences in adolescent UPF intake according to sex (Table 2). The most consumed UPFs according to their energy contribution to the diet were (1) sausage, burger meat, and chicken nuggets (16.8%); (2) regular bread, hot dogs, or burger bread (14.3%); and (3) carbonated or sweet beverages (14.0%). When comparing adolescents´ diets according to UPF intake, it was observed that in the UPF fraction, there was more than twice the proportion of kilocalories derived from simple sugars compared to the non-UPF fraction (25.9% vs. 11.5%, respectively; Table 2). Table 3 shows the kilocalories, macronutrients, and dietary fiber consumption by UPF intake quartiles in the participants. High UPF intake was associated (*p* < 0.001) with higher consumption of total kilocalories, total fat, and saturated fat, and lower intake of total carbohydrates and fiber density (Table 3).

**Table 2:** Energy and macronutrient contribution to the diet of ultra-processed foods and non-ultra-processed foods in adolescents from Antioquia.

Energy from diet macronutrients	Percentage of energy contribution of UPF	Percentage of energy contribution of non-UPF
Total energy intake (2025 kcal)	17.6%	82.4%
Protein intake (%)	7.5%	12.5%
Total fat intake (%)	32.3%	29.2%
Monounsaturated fat intake (%)	13.4%	9.3%
Polyunsaturated fat intake (%)	6.3%	7.4%
Total carbohydrate intake (%)	60.1%	58.2%
Free sugar intake (%)	25.9%	11.5%
Fiber density intake (g/1000 kcal)^a^	5.3 g/1000 kcal	13.2 g/1000 kcal
Energy by sociodemographic variables^b^		
Sex		
Male (*n* = 211)	17.2% (7.6–29.24)	82.8 % (70.8–91.4)
Female (*n* = 191)	19.0 % (8.4–29.7)	81.0 % (70.4–91.4)
Geographic area		
Urban (*n* = 359)	19.0 % (8.9–29.8)*	81.0 % (70.3–91.1)
Rural (*n* = 43)	9.7 % (1.1–23.8)	90.3 % (78.2–97.8)
Socioeconomic stratum		
Low (*n* = 335)	16.6 % (7.4–28.5)†	83.4% (71.7–92.6)
Medium (*n* = 67)	23.0 % (12.3–35.1)	77.0 % (65.1–87.1)

a Fiber density presented as grams of fiber intake for each 1,000 dietary kilocalories.

b Data are presented as median and interquartile range in parentheses.

UPF: Ultra-processed foods. *p < 0.01 Mann–Whitney U test for geographic area (urban vs. rural). †*p* < 0.01 Mann–Whitney U test for socioeconomic stratum (low vs. medium).

**Table 3:** Kilocalories, macronutrients, and dietary fiber consumption by ultra-processed food energy intake quartiles in adolescents from Antioquia.

Kilocalories/Nutrient	UPF intake quartile				*P*-value
	Quartile 1	Quartile 2	Quartile 3	Quartile 4	
Dietary energy intake (kcal/day)	1595.0 (1164.3–2096.7)	1874.2^a^ (1408.1–2345.4)	2075.4^a^ (1491.5–2778.6)	2089.1^a^^,^^b^ (1529.9–2769.1)	<0.001*
Protein (%)	11.8 ± 3.7	11.6 ± 3.1	11.7 ± 3.1	11.1 ± 3.2	0.417†
Total fat (%)	25.2 ± 8.8	28.9 ± 8.1^a^	31.9 ± 8.3^a^	33.6 ± 9.6^a^^,^^b^	**<0.001†**
Saturated fat (%)	10.0 (7.5–13.2)	10.5 (8.7–14.0)	12.3 (10.0–15.7)^a^^,^^b^	12.9 (10.0–16.0)^a^^,^^b^	<0.001*
Carbohydrates (%)	62.8 ± 10.0	59.4 ± 8.4^a^	56.3 ± 9.0^a^^,^^b^	55.1 ± 10.6^a^^,^^b^	**<0.001†**
Free sugars (%)	11.4 (6.9–15.0)	13.4 (8.9–19.6)	13.4 (8.6–17.3)	14.6 (8.3–18.1)	0.164*
Fiber density (g/1000 kcal)	6.8 (4.9–9.7)	5.9 (4.0–8.5)^a^	5.2 (4.1–7.0)^a^	4.6 (3.6–6.6)^a^^,^^b^	<0.001*

Data are presented as mean ± standard deviation or median and interquartile range in parentheses, according to the data distribution. Values represent the percentage of kilocalories provided by each macronutrient to the total dietary energy intake, unless otherwise indicated. Fiber density is presented as grams of fiber intake per 1,000 dietary kilocalories. Kcal: kilocalories, UFP: Ultra-processed foods. *p-value obtained by Kruskal–Wallis ANOVA (multiple comparison by Mann–Whitney U test). †*p*-value obtained by analysis of variance (multiple comparisons by Tukey’s test). Bold indicates statistically significant *p*-values (*p* < 0.05).

a Significant difference compared to quartile 1.

b Significant difference compared to quartile 2.

### Ultra-processed food product intake, nutritional status, and cardiovascular risk factors

Table 4 shows the association between UPF intake quartiles, nutritional status, blood lipids, and glucose concentrations in the participants. There was not a significant association between UPF intake and BMI, waist circumference, total cholesterol, triglycerides, HDL-C or LDL-C. However, adolescents from the fourth UPF intake quartile showed higher concentrations of blood glucose than those from the first quartile, 2.81 mg/dL and 3.13 mg/dL (*p* < 0.05), on crude and adjusted statistical models, respectively.

**Table 4:** Association between ultra-processed food energy intake quartiles and nutritional status variables, blood lipids, and glucose concentration in adolescents from Antioquia.

Variable	Quartile 1	Quartile 2			Quartile 3			Quartile 4		
		B	CI 95%	*p*-value	B	CI 95%	*p*-value	B	CI 95%	*p*-value
BMI										
Crude model	0	−0.25	−0.56 to 0.056	0.109	−0.09	−0.40 to 0.22	0.570	−0.21	−0.52 to 0.10	0.186
Adjusted model†	0	−0.20	−0.51 to 0.11	0.200	−0.06	−0.38 to 0.26	0.715	−0.17	−0.50 to 0.15	0.288
Waist circumference										
Crude model	0	−2.24	−4.69 to 0.21	0.064	−1.21	−3.66 to 1.25	0.362	−2.27	−4.74 to 0.20	0.140
Adjusted model†	0	−1.67	−3.96 to 0.62	0.152	−0.92	−3.25 to 1.42	0.441	−1.96	−4.32 to 0.41	0.105
Total cholesterol										
Crude model	0	−0.34	−9.88 to 9.20	0.985	−5.26	−14.80 to 4.28	0.310	−1.53	−11.16 to 8.11	0.644
Adjusted model†	0	−0.98	−10.52 to 8.57	0.841	−6.18	−15.91 to 3.55	0.213	−2.43	−12.29 to 7.44	0.629
LDL cholesterol										
Crude model	0	0.18	−7.60 to 7.60	0.805	−4.86	−12.63 to 2.91	0.159	−1.19	−9.04 to 6.66	0.443
Adjusted model†	0	−2.82	−11.27 to 5.63	0.513	−8.27	−16.88 to 0.34	0.060	−4.53	−13.27 to 4.20	0.308
HDL cholesterol										
Crude model	0	1.02	−1.78 to 3.82	0.526	2.13	−0.67 to 4.93	0.110	2.11	−0.72 to 4.94	0.111
Adjusted model†	0	1.0	−1.83 to 3.82	0.487	1.73	−1.14 to 4.61	0.237	1.79	−1.13 to 4.71	0.228
Triglycerides										
Crude model	0	0.36	−13.87 to 14.59	0.947	−0.82	−15.05 to 13.42	0.996	−0.35	−14.72 to 14.03	0.872
Adjusted model†	0	0.39	−14.19 to 14.96	0.959	1.30	−13.55 to 16.15	0.863	1.12	−13.95 to 16.18	0.884
Blood glucose										
Crude model	0	2.68	0.38 to 4.98	**0.023**	1.74	−0.56 to 4.05	0.137	2.81	0.50 to 5.13	**0.018**
Adjusted model†	0	3.14	0.84 to 5.44	**0.008**	2.12	−0.22 to 4.47	0.076	3.13	0.76 to 5.50	**0.010**

BMI: Body mass index, B: Beta coefficient, CI 95%: 95% confidence interval, LDL: Low-density lipoprotein, HDL: High-density lipoprotein. †Adjusted model for age in years, sex, socioeconomic stratum, geographic area, physical activity level, and total energy intake. Bold indicates statistically significant *p*-values (*p* < 0.05).

Table 5 shows the association between UPF intake quartiles and CRFs in adolescents from Antioquia. There was no significant association between UPF intake and excess body weight (overweight + obesity), abdominal obesity, or alterations in total cholesterol, triglyceride, or LDL-C levels. A positive association was observed between UPF intake and HDL-C level in the crude statistical model (*p* = 0.018); however, the statistical significance of the model disappeared after adjusting for sex, age, physical activity and sociodemographic variables (*p* = 0.118).

**Table 5:** Association between ultra-processed food energy intake quartiles and cardiovascular disease risk factors in adolescents from Antioquia.

Variable	Quartile 1	Quartile 2			Quartile 3			Quartile 4		
		OR	CI 95%	*p*-value	OR	CI 95%	*p*-value	OR	CI 95%	*p*-value
Excess body weight										
Crude model	1.0	0.74	0.38–1.46	0.391	0.66	0.33–1.32	0.236	0.72	0.36–1.43	0.352
Adjusted model†	1.0	0.77	0.38–1.55	0.467	0.69	0.33–1.43	0.314	0.72	0.35–1.50	0.379
Abdominal obesity										
Crude model	1.0	1.13	0.42–3.04	0.817	1.14	0.42–3.08	0.800	0.62	0.20–1.96	0.414
Adjusted model†	1.0	1.29	0.45–3.75	0.636	1.36	0.46–3.98	0.579	0.69	0.20–2.36	0.554
High total cholesterol										
Crude model	1.0	0.81	0.26–2.51	0.717	1.11	0.39–3.18	0.852	1.48	0.55–4.06	0.448
Adjusted model†	1.0	0.75	0.24–2.42	0.635	1.02	0.34–3.08	0.976	1.24	0.42–3.69	0.699
High LDL cholesterol										
Crude model	1.0	2.33	0.59–9.28	0.230	1.98	0.48–8.15	0.344	3.60	0.96–13.52	0.058
Adjusted model†	1.0	2.19	0.53–9.09	0.279	1.86	0.43–8.03	0.408	3.12	0.77–12.61	0.110
Low HDL cholesterol										
Crude model	1.0	0.76	0.43–1.34	0.338	0.61	0.34–1.09	0.093	0.48	0.27–0.88	**0.018**
Adjusted model†	1.0	0.86	0.46–1.60	0.626	0.72	0.38–1.36	0.309	0.59	0.30–1.14	0.118
High triglycerides										
Crude model	1.0	1.81	0.81–4.04	0.146	1.36	0.59–3.14	0.466	1.21	0.51–2.85	0.663
Adjusted model†	1.0	2.02	0.88–4.63	0.097	1.54	0.65–3.69	0.331	1.39	0.56–3.42	0.476

OR: Odds ratio, CI 95%: 95% confidence interval, LDL: Low-density lipoprotein, HDL: High-density lipoprotein. †Adjusted model for age in years, sex, socioeconomic stratum, geographic area, physical activity level, and total energy intake. Excess body weight classification was done by body mass index using the World Health Organization guidelines (22); high triglycerides and low HDL cholesterol were classified using the International Diabetes Federation criteria (23); and high total cholesterol, LDL cholesterol, and waist circumference (abdominal obesity) were classified according to the guidelines of the Expert Panel for Cardiovascular Health and Risk Reduction in Children and Adolescents (24). Bold indicates statistically significant *p*-values (*p* < 0.05).

## Discussion

The objective of this study was to analyze the association between UPF intake, diet quality, and CRFs in adolescents from Antioquia, Colombia. Higher UPF intake was found in adolescents from middle-class and urban areas of Antioquia; this may be due to the greater purchasing power of this segment of the population and greater availability of UPF in populated areas, as found in other studies (25, 26). The UPF consumption was associated with diet quality deterioration, characterized by higher intake of kilocalories and total and saturated fat and lower intake of dietary fiber. Additionally, a positive association was found between UPF intake and blood glucose levels in the participants. Nevertheless, there were no significant associations between UPF intake and CRFs in adolescents from Antioquia.

UPF consumption was associated with diet quality deterioration. Particularly, UPF consumption in adolescents showed positive associations with a higher intake of total kilocalories, total fat, and saturated fat and lower fiber intake. These results are probably due in part to the high caloric density of UPF, owing to the sugars and fats added to these products to improve their palatability (13, 27). Other factors that may have contributed to these results are the low UPF fiber amount, since, during the elaboration of these products, much of the natural food matrix is eliminated (13, 27). Similar results have been reported in other populations. Rauber et al., in children, adolescents, and adults from the United Kingdom, found that with higher UPF consumption, there was a higher intake of total fat and saturated fat (28). Viteri et al., in young people (14 to 17 years) from Ecuador, reported that high UPF consumption was associated with total fat and saturated fat intake over the nutrient recommendations of the Pan American Health Organization (29). A study by Neri et al., based on diet analysis from eight countries, reported a significant decrease in dietary fiber density in the higher UPF intake quintile (30).

The UPF intake was positively associated with blood glucose concentrations in adolescents from Antioquia. These results may be due in part to the high proportion of free sugars from UPF, since they contributed ~26% to the total UPF energy, which is more than double the proportion of kilocalories derived from free sugars in non-UPFs (Table 2). Similar findings have been reported by Khoury et al. in children from Spain, who reported a significant increase in blood glucose levels in the higher UPF intake tertile (31). The association between UPF intake and blood glucose, but not with blood lipids (or BMI), could represent metabolic adaptations in adolescents (i.e., increased insulin levels), which contribute to maintaining these biochemical markers within normal ranges at this relatively early age. Nevertheless, it cannot be ruled out that other factors not evaluated in the study, such as alcohol intake, drug and medication use, gut microbiota, diseases, and genetic factors, may have affected these findings. Noteworthy, it is important to consider that if adolescents from Antioquia, particularly those from urban areas and the middle class, continue with high UPF intake over time, they could develop metabolic alterations like hyperglycemia and dyslipidemia, as suggested by the studies of Lee et al. (32) and González-Palacios et al. (33).

There were no associations between UPF intake and CRFs in adolescents from Antioquia. These results are in line with some studies (34, 35), but in contradiction with others (36). The lack of UPF association with CRFs in this study may be due to the low amount of UPF consumed by adolescents, less than 18.0% of the total diet kilocalories (which is still high, given the recommendation to avoid such foods) (15, 37). Research showing positive associations between UPF and CRFs has reported higher consumption. For example, Canella et al., reported that UPF provided 25.5% of the total kilocalories (38), and in the study of Leffa et al., UPF provided 43.4% and 47.7% of the total kilocalories for children of different ages (39). An additional factor that may have contributed to the lack of association between UPF and CRFs in this study could be an underreported food intake of adolescents with excess body weight (and probably with higher CRFs), as observed in previous studies (40, 41).

This study expands the knowledge on a topic of great interest for public health, such as the association between UPF intake, diet quality, and CFRs in adolescents, given that this population group shows the highest UPF intake in Colombia and other countries (6–8, 42). To the best of our knowledge, this is the first study of Colombian adolescents to analyze the association between UPF intake and CRF, including high triglyceride, total cholesterol, LDL-C, blood glucose, and low HDL-C levels. Another strength of this study is the use of information from population surveys (Medellín Profile and Antioquia Profile), which collected data from representative samples of adolescents in the second most populous region in Colombia. Nonetheless, this study has limitations, as its cross-sectional design does not allow for the evaluation of causality or control for possible reverse causality. It is important to note that a period of exposure to UPF is required to observe the effects on the CRF analyzed here, which was not considered by the cross-sectional nature of the study. In addition, other factors such as alcohol intake, drug and medication use, gut microbiota, diseases, and genetic factors not evaluated in the study may have affected the findings. In addition, the use of 24-h dietary recall data may not be representative of the usual food intake by adolescents. However, this tool was used because it allows us to report precisely (grams or milliliters of) each food ingested, enabling a more precise quantitative diet analysis than the food frequency questionnaire commonly used in population studies.

In summary, UPF intake was related to diet quality deterioration and increased blood glucose concentrations in adolescents from Antioquia, Colombia. Chronic and continuous increases in blood glucose concentrations are known risk factors for cardiovascular diseases (CVD). However, CVD development is multifactorial and chronic, and has a long latency period. Therefore, further research is needed, particularly longitudinal studies that include data on physical activity, alcohol and tobacco consumption, sleep patterns, stress, and variables related to CVD development at an early age.

## Data Availability

The data analyzed in this study is subject to the following licenses/restrictions: data in custody by University Institution: Universidad de Antioquia and Gobernación de Antioquia. Requests to access these datasets should be directed to Universidad de Antioquia.
